# Lightning-generated waves detected at Mars

**DOI:** 10.1126/sciadv.aeb4898

**Published:** 2026-02-27

**Authors:** František Němec, Kateřina Rosická, Ivana Kolmašová, Ondřej Santolík

**Affiliations:** ^1^ Faculty of Mathematics and Physics, Charles University, Prague, Czech Republic.; ^2^ Department of Space Physics, Institute of Atmospheric Physics of the Czech Academy of Sciences, Prague, Czech Republic.

## Abstract

Although lightning activity has been confirmed on Jupiter, Saturn, and Neptune through the detection of lightning-generated electromagnetic waves, its occurrence on Venus and Mars remains unclear. Here, we report observations of such a frequency-dispersed whistler detected in the ionosphere of Mars by NASA’s MAVEN spacecraft. We demonstrate the plausibility of wave propagation from the atmosphere to the spacecraft, and we show that the observed dispersion corresponds to theoretical expectations using realistic crustal magnetic field and ionospheric models. Increased attenuation at higher frequencies explains why only the low-frequency part of the whistler is observed. Our observations provide direct evidence of electromagnetic waves from an impulsive source on Mars, suggesting that electric discharges may indeed occur in the Martian atmosphere.

## Teaser

Direct detection of a whistler on Mars shows that lightning-like discharges occur in its thin atmosphere.

## INTRODUCTION

Lightning-like discharges in the thin Martian atmosphere have not yet been observed (*1*), and their existence remains an open question. Simulations and laboratory experiments suggest that electric discharges are likely to occur in Martian dust storms similar to those observed in terrestrial volcanic eruptions and dust devils. During dust storms, dust grains become electrically charged through collisions. Using the known properties of Martian dust grains, the evolution of their charge density and electrostatic potential can be calculated (*2*). Numerical results indicate that, under specific conditions, the generated electric field can surpass the breakdown threshold in the low-pressure carbon dioxide atmosphere of Mars (*3*) (≈15 kV/m), resulting in a discharge. Recent simulations investigate dust grain parameters that control the strength of the generated electric field (*4*), demonstrating that peak electric fields may reach tens of kilovolt per meter. Laboratory experiments confirm the presence of visible electrical discharges in clouds of Martian regolith simulant under a low-pressure carbon dioxide atmosphere (*5*).

Terrestrial dust devils can generate ultralow-frequency radiation due to fluctuating charges in a convective vortex (*6*). Since Martian dust devils and dust storms are more frequent, stronger, and larger than those on Earth, theoretical studies suggest that they could generate nonthermal wideband radiation detectable from Earth (*7*). Radio observations of Mars from Earth have revealed unexpectedly strong microwave emissions in regions with substantial dust activity (*8*). Nevertheless, recent measurements by the Allen Telescope Array do not provide any evidence of Martian lightning (*9*). An extensive search for lightning-related wave intensification at frequencies in the range of 5 to 16 Hz, corresponding to Schuman resonances (*10*), in magnetic field data from the Mars Global Surveyor (MGS) and Mars Atmosphere and Volatile Evolution (MAVEN) missions has also been unsuccessful (*11*). Similarly, a search for impulsive radio signals, associated with lightning activity and penetrating the Martian ionosphere, in radar sounder data obtained by the Mars Express spacecraft has also yielded negative results (*1*).

**Fig. 1.**
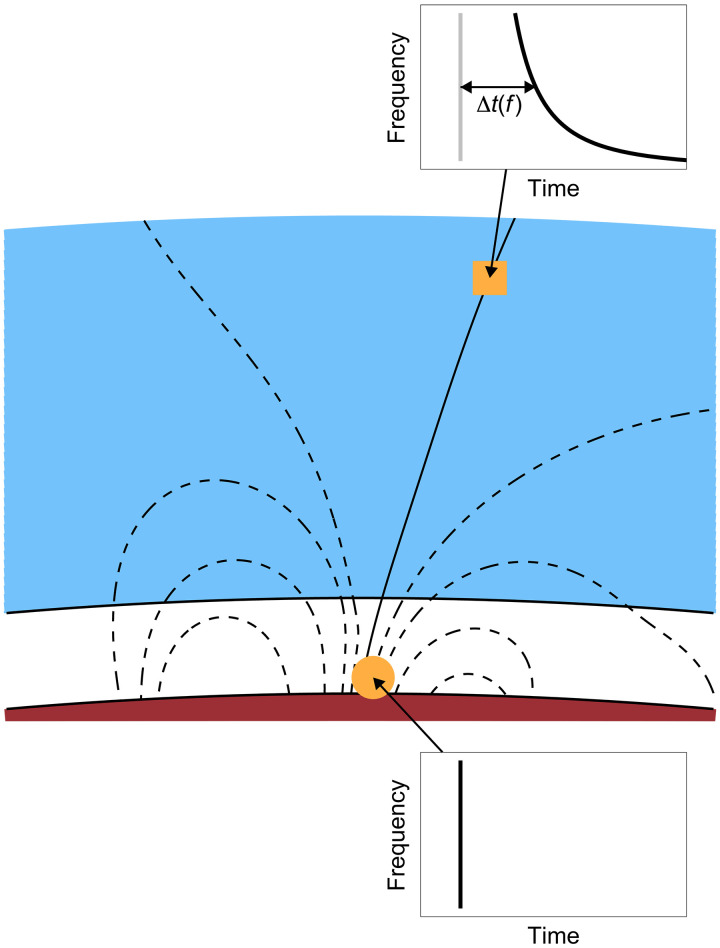
Schematic showing the formation of a whistler. A short electromagnetic pulse generated by an electric discharge (orange circle) spreads over time due to dispersion in the ionospheric plasma, where higher-frequency signals propagate faster than lower-frequency signals. These waves propagate to spacecraft altitudes (orange square) at locations where the magnetic field lines (black curves) are nearly vertical.

Another possibility for detecting electric discharges stems from the analysis of accompanying electromagnetic radiation in the extremely low frequency/very low frequency range, which, under favorable conditions, can also penetrate the ionosphere. First established and understood on Earth shortly before the space era (*12*), these waves have been successfully used to evidence lightning occurrence and properties on Jupiter (*13*–*16*), Saturn (*17*), and Neptune (*18*). These waves, characterized by a distinct spectral pattern resulting from frequency dispersion in the plasma medium, are known as whistlers. A sketch of their formation is shown in Fig. 1. A short broadband pulse of electromagnetic radiation is generated during a lightning discharge (orange circle, spectral shape at the bottom). However, the ionospheric plasma (blue region) is dispersive, with higher frequencies propagating faster and arriving at the observation point sooner than lower frequencies. This results in a characteristic “whistling” spectral shape (*19*) (Fig. 1, top) for wave frequencies lower than one quarter of the electron cyclotron frequency, which is typically the case. Note that whistler-mode waves cannot propagate at frequencies above the electron cyclotron frequency. Moreover, they can effectively penetrate the Martian ionosphere only on the nightside and when the magnetic field has a notable vertical component (*20*, *21*). This largely restricts the areas over Mars where whistlers could be observed by spacecraft to the relatively small crustal field regions in the southern hemisphere.

In this study, we search for possible whistlers detected by the MAVEN spacecraft (*22*), which has been orbiting Mars since 2014. Wave data spanning sufficiently high frequencies are extremely sparse, consisting of 1-s-long snapshots of a single electric field component sampled at 1024 Hz, available only for selected time intervals chosen based on the waveform amplitudes (*23*). Using the Fourier transform, we calculate the frequency time dependence of the wave intensity for each snapshot. Considering that potential lightning-generated waves are more likely to be observed at lower altitudes, we focus on measurements taken at spacecraft altitudes below 500 km. Visual inspection of all corresponding wave snapshots (about 100,000) results in a single snapshot containing a possible whistler.

## RESULTS

### Observations

**Fig. 2.**
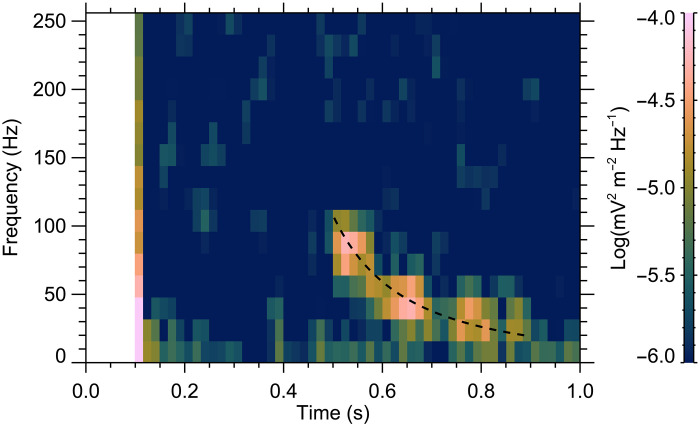
Frequency-time spectrogram of electromagnetic waves measured by MAVEN. Wave intensity is color-coded according to the scale on the right. A dispersed whistler is observed in the second half of the time interval at frequencies up to about 110 Hz. The black dashed line shows a theoretical spectral shape for an impulsive source in the Martian atmosphere, calculated from a model with realistic magnetic field and plasma density profiles.

Frequency-time spectrogram corresponding to the identified whistler is shown in Fig. 2. The respective wave snapshot was taken on 21 June 2015 at 12:47:27 universal time (UT). At the time of the observation, the spacecraft was at an altitude of 349 km, at a longitude of −125.8° and a latitude of −73.4°, above the region of substantial crustal magnetic fields. The magnetic field measured by the spacecraft was nearly vertical, with an inclination of −80.6° and a magnitude of about 40.3 nT, corresponding to the electron cyclotron frequency of about 1128 Hz. The solar zenith angle (SZA) of the observation was about 103.7°, just beyond the terminator. Starting in the second half of the plotted time interval, the whistler lasts for about 0.4 s. The wave frequency gradually decreases with time, corresponding to the expected whistler spectral shape. While the available wave measurements cover the frequencies up to 512 Hz, only the lower half of the frequency interval is plotted, as the whistler itself spans frequencies up to only about 110 Hz. The power spectral density of the whistler is ~2 × 10^−5^ mV^2^ m^−2^ Hz^−1^, about an order of magnitude stronger than the background noise in this wave snapshot. No exceptionally strong dust activity is identified at the given location and time, according to the available climatology of the Martian atmospheric dust optical depth (*24*, *25*).

### Whistler dispersion

Theoretical calculations of the whistler dispersion are used to verify that the observed whistler-like emission indeed originates from a short, wideband electromagnetic pulse at low atmospheric altitudes. In the plasma medium, whistler-mode waves tend to propagate along magnetic field lines (*26*), even more so when ducted by density inhomogeneities aligned with those lines (*27*–*29*). The speed of wave propagation depends on the wave frequency, magnetic field magnitude, and plasma density, and it can be notably lower than the speed of light. To obtain a theoretical whistler shape, we trace the magnetic field line from the spacecraft location down to the Martian surface and evaluate the corresponding time delays of wave propagation as a function of frequency. In the neutral atmosphere below the ionospheric bottom, the signal propagates in free-space mode rather than along the magnetic field. This segment of the path contributes only a negligible, frequency-independent delay, and the resulting deviation from the field line does not affect the analysis.

**Fig. 3.**
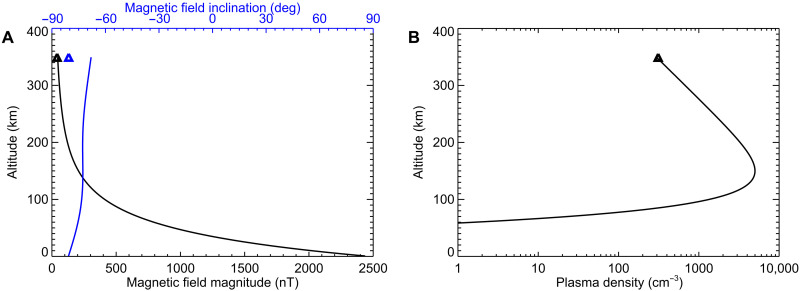
Altitude profiles of magnetic field and plasma density. (**A**) Altitude profiles of the magnetic field magnitude (black) and magnetic field inclination (blue, the angle with respect to the horizontal) corresponding to a crustal magnetic field model. (**B**) Altitude profile of plasma density. The triangle symbols at the highest altitude (about 350 km) indicate the values measured at the spacecraft location.

Considering that the event takes place beyond the terminator and in a strong crustal magnetic field region, external magnetic fields formed due to electric currents in the near-Mars outer space (*30*, *31*) are neglected. An empirical crustal magnetic field model (*32*), parameterized exclusively by location, is then used to provide the magnetic field vector. The altitude profiles of the magnetic field magnitude (black) and inclination with respect to the horizontal plane (blue), calculated along the magnetic field line passing through the spacecraft, are shown in Fig. 3A. The field line remains almost vertical all the way down to the planetary surface, while the magnetic field magnitude gradually increases. The colored triangle symbols at the highest altitude correspond to MAVEN magnetic field measurements (*33*) at the time of the event, showing reasonable agreement with the model, even slightly closer to the local vertical direction.

The dayside plasma density profile around the ionospheric peak can be approximated by a single Chapman layer (*34*). While the situation on the nightside is more complicated and the ionosphere may be patchy or slab-like, our event takes place just beyond the terminator, where the ionosphere is still rather day-like due to day-night transport and residual photoionization (*35*, *36*). Considering typical parameters based on many ionospheric sounding (*37*) and radio occultation (*38*) observations, the following Chapman parameters appear reasonable for the SZA of the observation: peak altitude of 150 km, peak plasma density of 5000 cm^−3^, and scale height of 30 km. The resulting plasma density profile is shown, along with the in situ measured plasma density at the spacecraft altitude, in Fig. 3B.

Assuming these magnetic and plasma density profiles, the time delays due to wave propagation can be calculated using the known plasma dispersion relation (*19*, *39*). The resulting spectral shape is plotted by the dashed curve in Fig. 2, agreeing well with the observations. While the propagation from the surface to the spacecraft takes about 0.32 s for a wave with a frequency of 110 Hz, a wave with a frequency of 20 Hz propagates for ~0.71 s.

### Propagation through the ionosphere

Theoretical calculations can further help us demonstrate the feasibility of electromagnetic wave propagation from low atmospheric altitudes to the spacecraft and, at least qualitatively, understand the frequency range of the observed whistler, particularly its upper frequency cutoff. Its frequency of about 110 Hz is well below the electron cyclotron frequency, which is the theoretical upper-frequency limit for whistler-mode wave propagation in a magnetized plasma (*26*). However, wave attenuation due to the collisional ionospheric medium must be considered. This attenuation depends on the altitude profiles of the collisional frequencies, which, in turn, can be obtained from altitude profiles of neutral density and electron temperature through empirical relations (*20*, *40*).

**Fig. 4.**
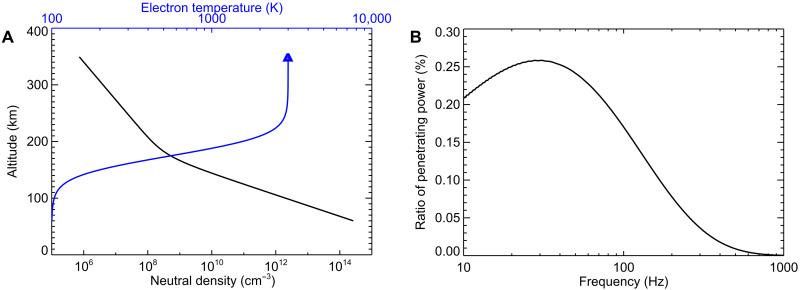
Wave attenuation during propagation through the ionosphere. (**A**) Altitude profiles of neutral density (black) and electron temperature (blue) corresponding to typical values in the Martian atmosphere, used for the calculation. (**B**) Calculated ratio of wave power penetrating from the ground to the spacecraft as a function of the wave frequency.

Although subject to considerable uncertainties, we assume realistic neutral density and electron temperature profiles depicted in Fig. 4A, which are consistent with former observational and modeling efforts (*41*–*44*) and with the measured electron temperature (*23*) (neutral density measurements are not available for the time of the event). We follow an established full-wave modeling method (*45*, *46*) for the electromagnetic field of the propagating waves. Figure 4B shows the resulting ratio of power penetrating from the anticipated source close to the planet to the spacecraft altitude as a function of frequency. While waves with frequencies below about 100 Hz can reach the spacecraft with notable attenuation, higher-frequency waves are attenuated even more, consistent with the upper-frequency limit of the observed whistler. This modeling method also confirms the above-described whistler dispersion calculations. Last, we note that although considering different altitude profiles of the controlling parameters would yield slightly different quantitative outputs, the results would not differ qualitatively and would remain consistent with the observations (see the parameter variation analysis in figs. S1 to S5).

## DISCUSSION

Lightning-like discharges have been shown to occur in the atmospheres of several Solar System planets, but direct observational evidence of their presence on Mars was still missing. We present a whistler event detected by the MAVEN spacecraft in the ionosphere of Mars, whose spectral shape closely resembles that of well-known lightning-generated whistlers on Earth. The dispersion of the event is consistent with calculations based on realistic magnetic field and plasma density profiles. Numerical calculations of wave attenuation during propagation from the atmosphere to the spacecraft demonstrate that propagation from a sufficiently intense impulsive source is indeed viable, facilitated by the weak ionosphere beyond the terminator and the strong, almost vertical crustal magnetic field in the analyzed region. These calculations further explain that it is the attenuation in the ionosphere that prevents the whistler from being observed at higher frequencies.

Despite the large number of MAVEN-measured wave snapshots investigated (108,418 in total), only a single event was identified. It occurred on the nightside, in a region with a nearly vertical magnetic field, which is a necessary condition for the successful wave propagation to higher ionospheric altitudes (*21*). We note that while nightside ionospheric conditions were present in about one-third of the analyzed wave snapshots, these high magnetic field inclinations are extremely rare; fewer than 1% of the investigated wave snapshots (679 in total) were measured at locations with these high values, and only 290 of them at SZA > 100°. This suggests that although lightning-like electric discharge processes can occur on Mars, the ionospheric properties often preclude the formation of a detectable whistler. In addition, the discharges themselves may be infrequent or weak, possibly due to additional processes hindering breakdown electric field generation (*47*).

We also note that other natural plasma waves generated by plasma instabilities are occasionally present in the Martian ionosphere and detected by MAVEN (*48*, *49*). These may have considerable intensities, and some even exhibit complex frequency-time structures resembling chorus emissions at Earth. However, they are markedly different from discharge-generated whistlers (see examples in fig. S6), as they are generally composed of repeating rising tones with high-frequency sweep rates (*49*) and are confined to mini-magnetosphere–like regions with closed magnetic field lines (*50*). The whistler event we report is thus distinguished by both its characteristic spectral shape and the exceptional location of a nearly vertical magnetic field. Interpreting it as a whistler propagating from an electrical discharge is the most plausible explanation, fully consistent with the observations. The observed whistler power spectral density corresponds to a frequency-integrated amplitude of ~0.04 mV/m. Although this is somewhat weaker than typical whistler amplitudes at Earth (*51*, *52*), accounting for the refractive index at the spacecraft location and for ionospheric attenuation yields an estimated Poynting flux on the ground of about 0.5 μW/m^2^, which would correspond to a strong terrestrial event (*53*). Note that we cannot determine the location where the discharge occurred. It may have taken place in the atmosphere, but more likely in a regional dust storm, a dust devil, or perhaps through some other yet-unknown process. We also cannot identify its exact location on the planet to within a few hundred kilometers due to the possible signal propagation in the Mars-ionosphere waveguide (*54*). In summary, this study presents an observation of a whistler event on Mars, indicating lightning-like electrical discharge activity.
