# Supplementary material for: Lightning-generated waves detected at Mars
Source: Sci Adv. 2026 Feb 27;12(9):eaeb4898. doi: 10.1126/sciadv.aeb4898 (PMC12947858; doi:10.1126/sciadv.aeb4898)
Supplement: Supplementary file 1 — Figs. S1 to S6 [file sciadv.aeb4898_sm.pdf]

Science Advances
AAAS

Supplementary Materials for

Lightning-generated waves detected at Mars

František Němec *et al.*

Corresponding author: František Němec, frantisek.nemec@mff.cuni.cz

Sci. Adv. **12**, eaeb4898 (2026)
DOI: 10.1126/sciadv.aeb4898

This PDF file includes:

Figs. S1 to S6

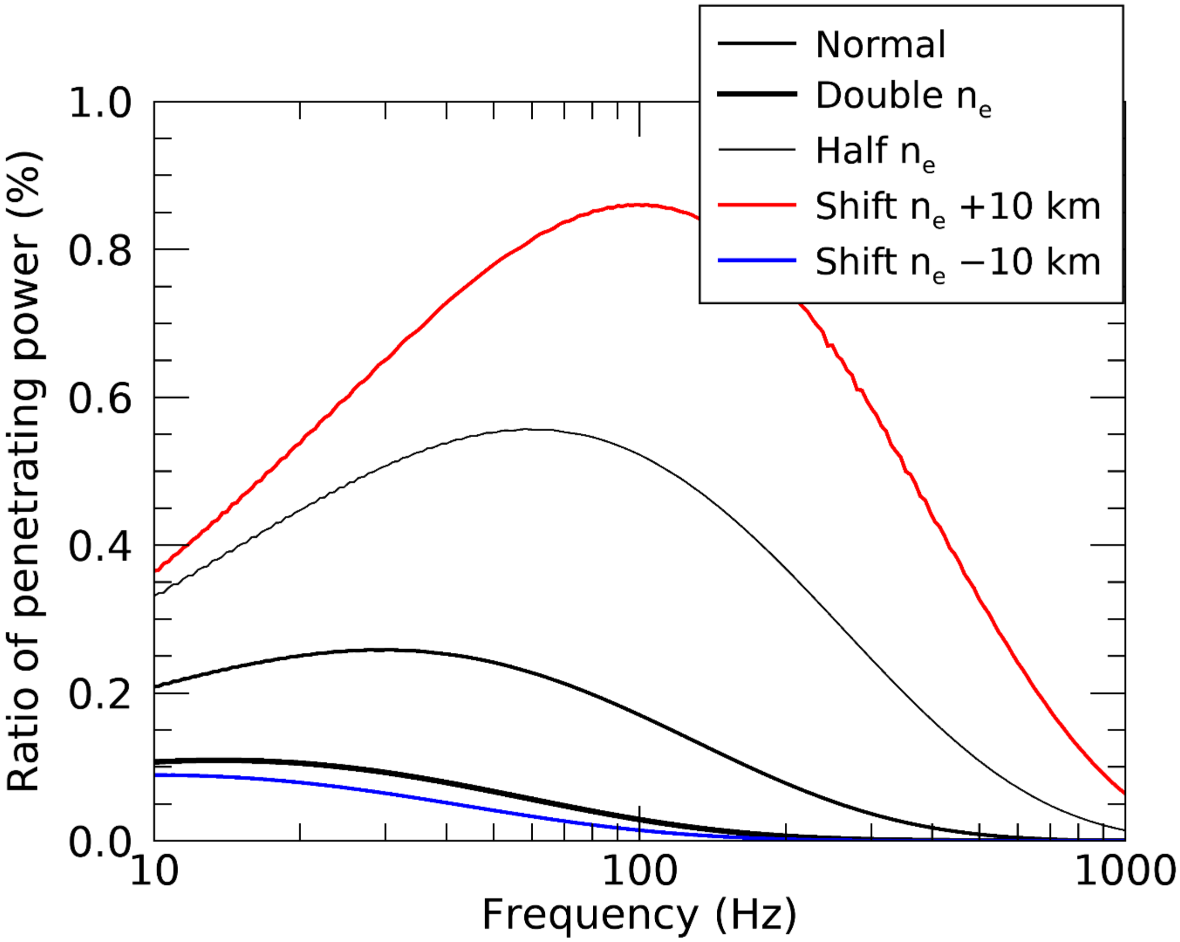

Fig. S1. Attenuation spectra calculated for modified plasma density profiles. Results for five different plasma density profiles are shown, as indicated in the legend: the normal plasma density profile considered in the paper, a profile with double the plasma density, a profile with half the plasma density, a profile shifted 10 km upward in altitude, and a profile shifted 10 km downward in altitude. Increasing the plasma density or shifting the profile downward both result in stronger attenuation, whereas decreasing the plasma density or shifting the profile upward results in weaker attenuation.

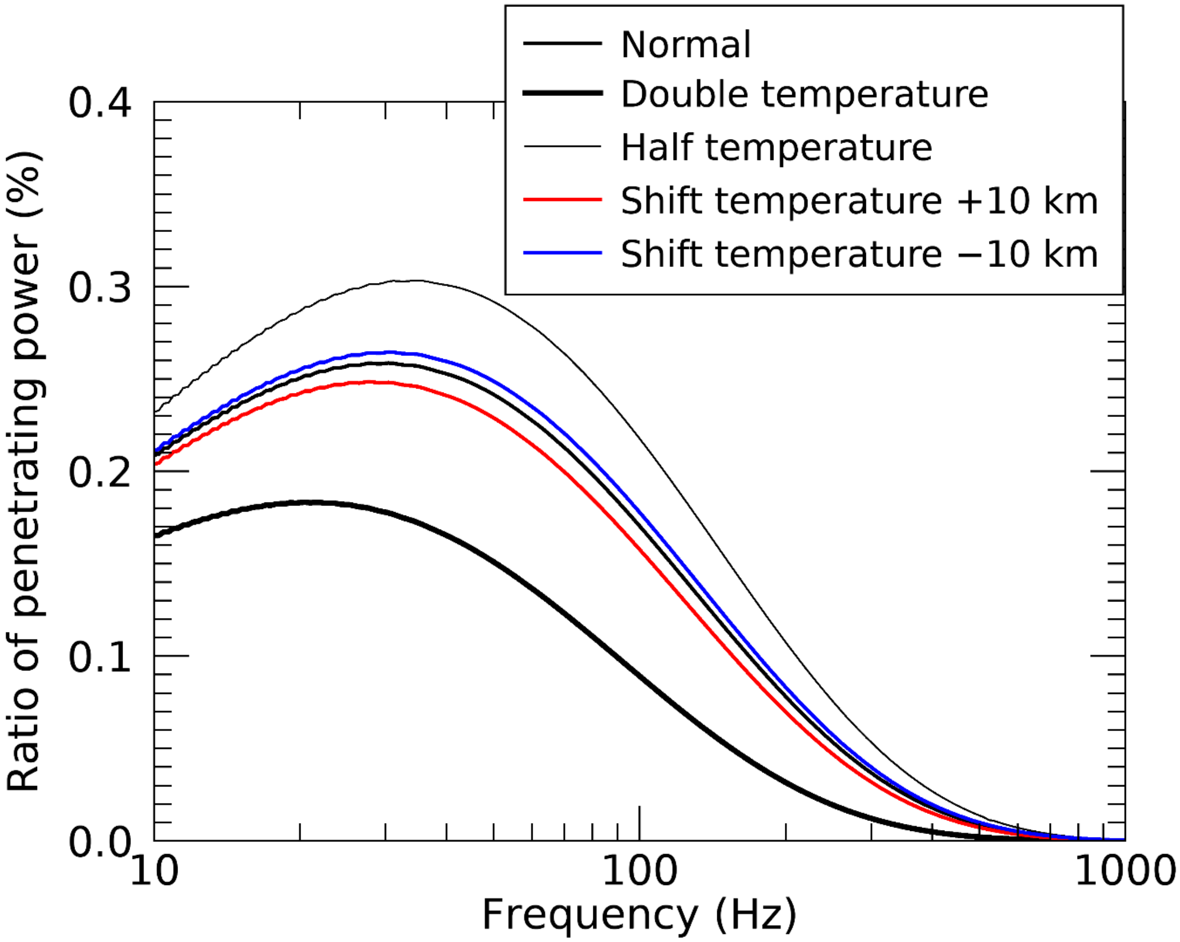

Fig. S2. Attenuation spectra calculated for modified electron temperature profiles. Results for five different electron temperature profiles are shown, as indicated in the legend: the normal electron temperature profile considered in the paper, a profile with double the electron temperature, a profile with half the electron temperature, a profile shifted 10 km upward in altitude, and a profile shifted 10 km downward in altitude. Increasing the electron temperature results in stronger attenuation, whereas decreasing the electron temperature results in weaker attenuation.

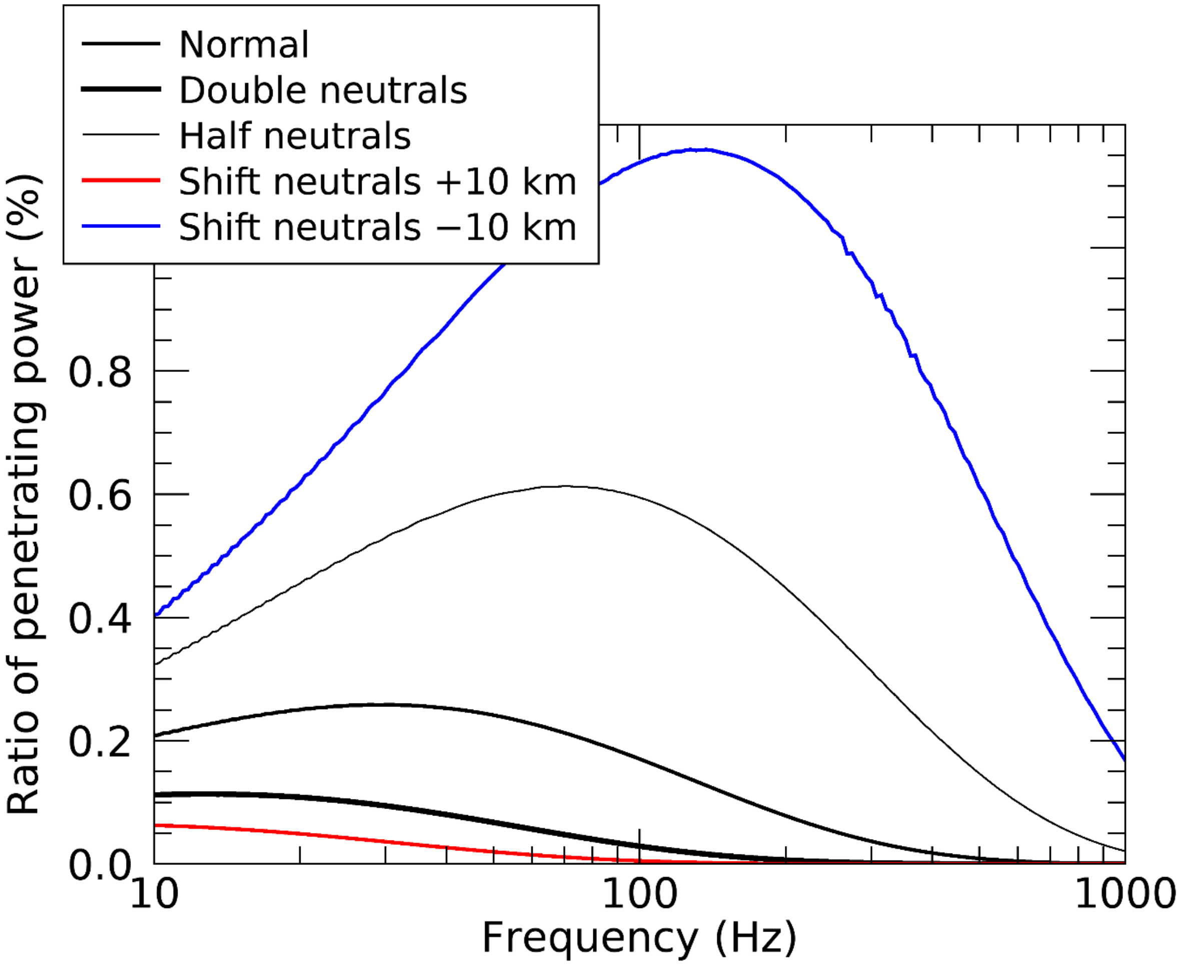

Fig. S3. Attenuation spectra calculated for modified neutral density profiles. Results for five different neutral density profiles are shown, as indicated in the legend: the normal neutral density profile considered in the paper, a profile with double the neutral temperature, a profile with half the neutral temperature, a profile shifted 10 km upward in altitude, and a profile shifted 10 km downward in altitude. Increasing the neutral density or shifting the profile upward both result in stronger attenuation, whereas decreasing the neutral density or shifting the profile downward results in weaker attenuation.

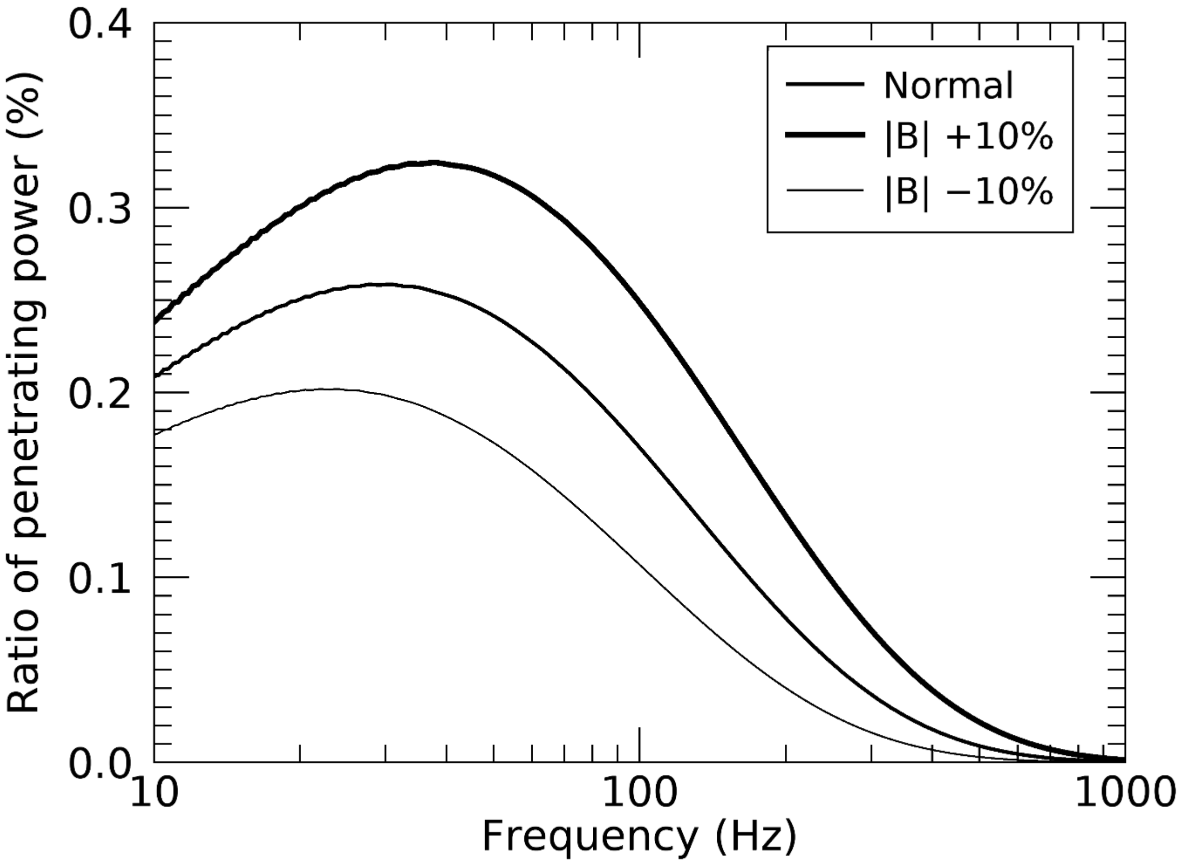

Fig. S4. Attenuation spectra calculated for modified magnetic field magnitude profiles. Results for three different magnetic field magnitude profiles are shown, as indicated in the legend: the normal magnetic field magnitude profile considered in the paper, a profile with the magnetic field magnitude increased by 10%, and a profile with the magnetic field magnitude decreased by 10%. Increasing the magnetic field magnitude results in weaker attenuation, whereas decreasing the magnetic field magnitude results in stronger attenuation.

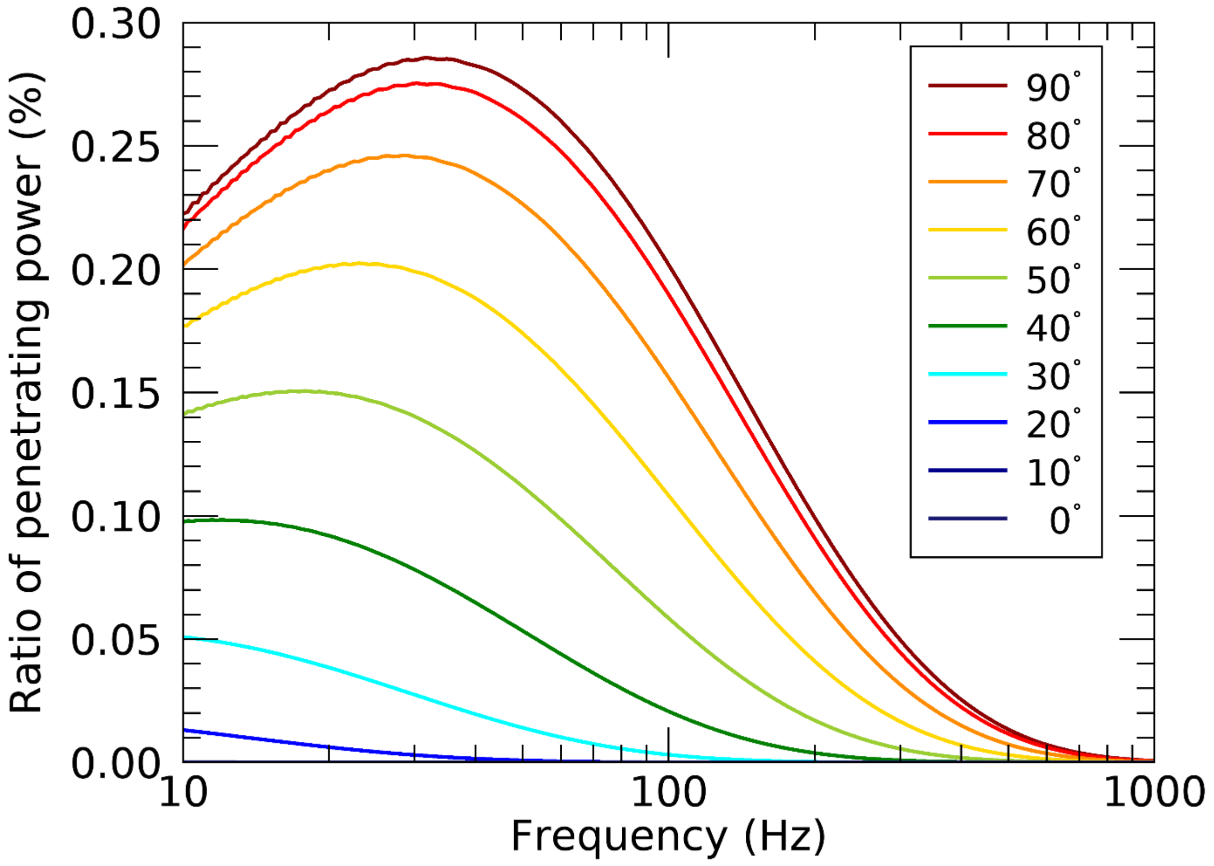

Fig. S5. Attenuation spectra calculated for different magnetic field inclinations. An inclination of 0° corresponds to a horizontal magnetic field orientation, whereas an inclination of 90° corresponds to a vertical magnetic field orientation. Results for various magnetic field inclinations are shown, as indicated in the legend. The ratios of penetrating power for magnetic field inclinations of 0° and 10° are essentially zero and therefore are not shown in the graph. Increasing the magnetic field inclination results in weaker attenuation, whereas decreasing the magnetic field inclination results in stronger attenuation.

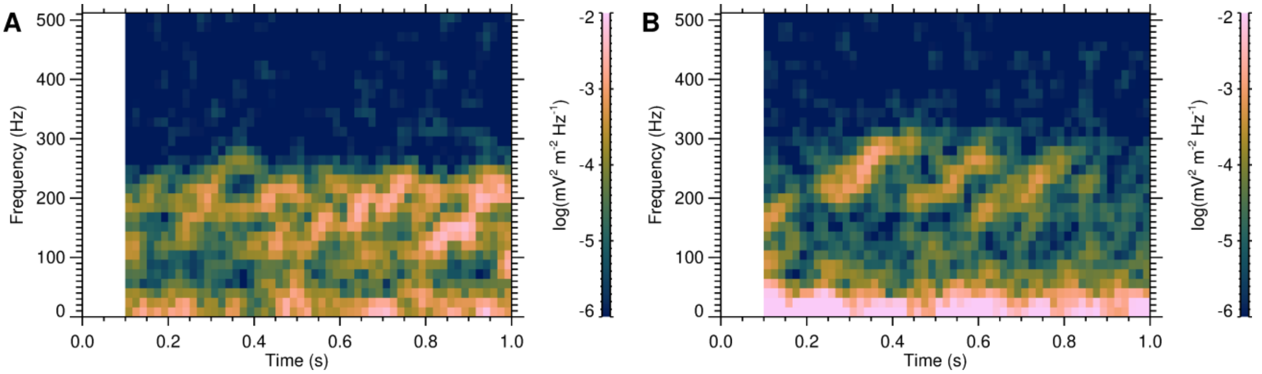

Fig. S6. Examples of frequency-time spectrograms of emissions with complex frequency-time structures. These emissions, measured by MAVEN, may take the form of banded emissions or even consist of repeating rising tones with high frequency sweep rates that resemble terrestrial chorus emissions. **(A)** Event measured on 2015/07/12 at 06:02:29 UT. **(B)** Event measured on 2015/07/15 at 08:17:31 UT.
